# High Content of Thermoplastic Starch, Poly(butylenes adipate-co-terephthalate) and Poly(butylene succinate) Ternary Blends with a Good Balance in Strength and Toughness

**DOI:** 10.3390/polym15092040

**Published:** 2023-04-25

**Authors:** Zhaoyang Niu, Fangping Chen, He Zhang, Changsheng Liu

**Affiliations:** 1 Engineering Research Center for Biomedical Materials of Ministry of Education, East China University of Science and Technology, Shanghai 200237, China; oasis123nirvana@163.com (Z.N.); m15868505633@163.com (H.Z.); 2 Key Laboratory for Ultrafine Materials of Ministry of Education, School of Materials Science and Engineering, East China University of Science and Technology, Shanghai 200237, China

**Keywords:** high starch content, thermoplastic starch, poly(butylene succinate), poly(butylenes adipate-co-terephthalate)

## Abstract

The ternary blends of a high content of thermoplastic starch (TPS), poly(butylenes adipate-co-terephthalate) (PBAT), and poly(butylene succinate) (PBS) were first melt-compounded in a twin screw extruder. The TPS contents in ternary blends were fixed at 60 wt%. The miscibility, morphology, thermal behavior, mechanical properties, and thermal resistance of the blends were investigated. The results showed that dispersions of PBS and PBAT minor phases improved the tensile strength and elongation at break. TPS/PBS/PBAT60/10/30 formed a good balance in strength and toughness. Dynamic mechanical analysis of the blends exhibits an intermediate and peak suggesting the ternary blend is compatible. Minor phase-separated structure SEM results showed that TPS/PBS/PBAT60/10/30 blend formed a typical mixture with core−shell morphology. As the PBAT composition was increased, phase morphology changes occurred in the blends, leading to decreased values of complex viscosity, storage modulus, and loss modulus. Moreover, the thermal resistances and melt flow properties of the materials were also studied by analysis of the heat deflection temperature (HDT) and melt flow index (MFI) value in the work.

## 1. Introduction

In recent years, due to the rapid development of electronic commerce, a large number of disposable packaging materials such as express packaging and fast food packaging are used and discarded, causing a great burden on the natural environment. Although governments have introduced a series of plastic ban policies, the implementation of the plastic ban policy is still faced with great resistance due to the high cost of biodegradable materials and their performance cannot meet the application needs in most scenarios. It is of great application significance to develop a biodegradable material with excellent performance and low cost. Starch is one of the promising biodegradable raw materials with its abundant availability, competitive price and renewable properties [1]. By adding plasticizers such as water and glycerin, starch can be transformed into thermoplastic starch (TPS) with certain processing properties under the action of heating and shearing [2]. However, pure thermoplastic starch has high hydrophilicity and poor mechanical property compared with other thermoplastic polymers, which hindered its application. To overcome these shortcomings, blending other biopolymers with TPS represent an alternative method to prepare biodegradable bioplastics [3,4,5]. 

As an environmentally friendly and energy-efficient synthetic aliphatic polyester, Poly(butylenes adipate-co-terephthalate) (PBS) possesses excellent processability, mechanical properties, biodegradability and thermal stability [6,7]. To enhance the mechanical properties and decrease the cost of PBS bioplastics, the introduction of TPS to PBS is an economical and effective method [8,9]. Yun et al. found that PBS/TPS blends with a mass ratio of 80/20 exhibited a good balance in strength and toughness, which is conducive to its application in food packaging [10]. Liu et al. prepared PBS/TPS blends with a weight fraction ratio of 80/20 and higher interfacial compatibility of the blends was obtained with the role of the ionic liquid plasticized starch [11]. Although TPS/PBS blends have been variously studied, the contents of TPS tend to be limited between 10% and 20%. Meanwhile, the mechanical properties of the TPS/PBS blends decline sharply with the increase in TPS content. The cost advantage of TPS components can hardly compensate for the performance loss of TPS blends.

Our research group developed a high content of TPS/degradable polyester melt blend method. Even when the weight fraction of TPS reached about 60%, the properties of the blend had potent mechanical properties [12]. We prepared a series of TPS/PBS (60/40) melt blends and found that the mechanical properties of the blends improved with the increased content of compatibilizers. When the weight fraction of the compatibilizers was 10%, the tensile strength of the blends exceeded 9 MPa. With further optimization of the process, the tensile strength of the TPS/PBS blends prepared by our research group could be stabilized at about 15 MPa. However, due to the inherent rigidity of the TPS and PBS components, the elongation at the break of the blends is less than 5%. It is difficult to meet the flexibility requirements of packaging materials.

Compared to binary blends, ternary or multi-component polymer materials usually show more excellent mechanical properties [13,14]. The introduction of a third component in TPS/PBS blends is expected to result in the flexibility problem, thus realizing the application of low-cost TPS degradable materials in packaging materials. As the first choice for biodegradable film materials at present, PBAT is a copolymer of butylene glycol adipate and butylene terephthalate, which has excellent ductility and elongation at break, good heat resistance and impact properties [15]. Currently, studies have been conducted to achieve the balance between rigidity and toughness of binary or ternary blends by using PBAT as a toughening agent. Aldas prepared Gum rosin plasticized PLA/PBAT (80/20) blend and found that Gum rosin could adjust PBAT domain size. When the weight fraction of Gum rosin reached 15% wt., the impact strength of the PLA/PBAT blend was 80% higher than that of pure PLA [16]. Ren et al. prepared TPS/PLA/PBAT ternary blends by the one-step extrusion method and revealed that the addition of PBAT significantly improved the impact strength and elongation at the break of the TPS/PLA matrix [17]. Therefore, under the condition of ensuring good interface compatibility between TPS and PBS, the addition of PBAT components is expected to form a core-shell structure and improve the flexibility of TPS/PBS blends. 

To the best of our knowledge, the ternary blends of TPS, PBAT and PBS have been studied very little so far. Considering the promising complementary properties among TPS, PBAT and PBS blend components, In the present work, we focus on the ternary blends with TPS, PBAT and PBS by simple melt blending method to obtain high starch-content bioplastic blends with excellent tensile strength and elongation at break. TPS was used as a matrix, respectively, whereas PBS and PBAT were used as minor phases in the blends. The miscibility, crystallization behavior, phase morphology, mechanical properties, and thermal resistance of the blends system were investigated.

## 2. Experimental

### 2.1. Materials

Cassava starch with 12 wt% moisture content was provided by Shengda Co. (Lanzhou, China). PBAT, with weight-average molecular weight (Mw) of 15.5 × 10^4^ g/mol and polydispersity index (PI) of 1.62, was purchased from Xinfu Co. (Hangzhou, China). PBS (Mw = 1.4 × 10^5^ g/mol and PI = 1.82) was supplied by Wuhu Plastics Co. (Wuhu, China). Glycerol was from Sino Chemical Reagent Co. (Shanghai, China). Maleic anhydride (MAH) was from Lingfeng Chemical Reagent Co. (Shanghai, China) and Dicumyl peroxide (DCP) was from Aladdin Bio-Chem Technology Co. (Shanghai, China).

**Figure 1:**
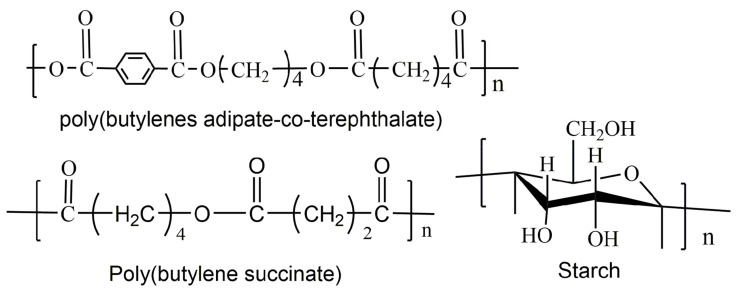
Chemical structures of PBAT, PBS and starch.

The chemical structures of the PBAT, PBS and cassava starch for the blends are illustrated in Figure 1. All the chemicals used in this study had a purity level of reagent grade unless otherwise noted.

### 2.2. Preparation of TPS 

Cassava starch, water and glycerol were mixed in a high-speed mixer for 5 min and then sealed for 24 h. TPS was extruded through a co-rotating twin screw extruder (SHJ-20, Nanjing, China) with an L/D ratio of 46. The temperature profiles from feed throat to die were 85 °C, 90 °C, 95 °C, 95 °C, 100 °C, 100 °C, 105 °C and 105 °C. The screw speed was 200 rpm and the feed speed was 0.2 kg/h, respectively. The extruded strips of TPS were cut into small cylinders.

### 2.3. Synthesis of PBAT-g-MA

PBAT, MAH and DCP were sequentially added to the HAKKE rheometer in a mass ratio of 200/8/1. The temperature of the rheometer was set to 145 °C and the screw speed was 60 rpm. PBAT-g-MA with excellent interface compatibilization was obtained when the rheometer torque was stable.

### 2.4. Preparation of the Blends

After TPS, PBAT, PBS and PBAT-g-MA were dried at 80 °C in the oven for at least 4 h, the blends were compounded in a co-rotating twin screw extruder. The temperature profiles from feed throat to die were 115 °C, 120 °C, 125 °C, 125 °C, 130 °C, 135 °C, 140 °C and 140 °C. The screw speed was 200 rpm and the feed speed was 0.2kg/h. The molten blends were transferred from the extruder to a preheated small injection mold for further evaluation. Samples with different component contents were prepared by changing the mass ratio of TPS, PBS and PBAT in the blend and fixing the mass of PBAT-g-MA to 5% of the sum of the mass of PBAT and PBS.

### 2.5. Mechanical Properties

Tensile strength and elongation at break were measured with a mechanical tensile tester (CMT6104-SANS, Shenzhen, China) according to ASTM D638. Crosshead speed was 10 mm/min. Each sample was tested five times and the average value was calculated. All samples were conditioned at 25 °C for 72 h under a relative humidity of 50%.

### 2.6. Differential Scanning Calorimetry (DSC)

The cold crystallization temperature (T_c_), and melting temperature (T_m_) of the blends were characterized on a TA Q200 DSC instrument under a N_2_ atmosphere. For each measurement of thermal behavior, all the samples were initially heated from 25 °C to 150 °C at a heating rate of 10 °C/min, kept at 150 °C for 3 min to eliminate the thermal history, and then cooled to −50 °C at a cooling rate of 5 °C/min. Subsequently, a second heating scan was conducted from −50 °C to 150 °C at the same ramp of 10 °C/min, taking the data from the second heating.

### 2.7. Dynamic Mechanical Analysis (DMA)

A dynamic mechanical analyzer (U.B.M, RheleogyE4000, Japan) was adopted to determine the storage modulus and Tan delta of blends. The experiment was operated under a tension mode, at an oscillating frequency of 1 Hz. The samples were scanned as a function of temperature from −60 °C to 100 °C at a constant heating rate of 3 °C/min.

### 2.8. X-ray Diffraction (XRD)

Crystal structure and type of the blends were analyzed by D/max 3-V X-ray diffraction (Rigaku, Tokyo, Japan), operated at 40 kV and 100 mA using Cu-Ka1 radiation. The scattering angle 2Ɵ covered the range from 5° to 50° with a step of 0.02° and at a scanning speed of 3°/min. 

### 2.9. Rheological Evaluation

The rheological properties (Storage modulus, loss modulus, complex viscosity) of the blends were observed by an AR 2000 rheometer from TA Instruments. Disk-shaped samples of 25 mm diameter and 1 mm thickness were measured at 140 °C with a gap opening of 1.2 mm. Dynamic properties were determined by a dynamic frequency sweep test. The range of frequency was 0.01 HZ to 100 HZ, and the strain was kept at 1%. These limits were fixed based on the polymer torque sensitivity and thermal stability.

### 2.10. Scanning Electron Microscope (SEM)

To obtain the natural fracture surface, samples were kept in liquid nitrogen to break the interfacial adhesion; the morphology of the samples of the blends were observed with scanning electronic microscopy (SEM, Hitachi H-800, Tokyo, Japan) at an acceleration voltage of 15 kV. Samples were coated with gold for 40 s. 

### 2.11. Thermogravimetric Analysis (TGA)

The thermal stability of samples was determined by a Q5000 instrument (TA instruments, New Castle, DE, USA). The samples were placed in an aluminum pan and heated from 25 °C to 600 °C at 10 °C/min under nitrogen.

### 2.12. Heat Deflection Temperature (HDT)

HDT was performed according to ASTM D648. The same instrument and samples for DMA were applied for HDT measurements in three-point bending mode at an applied load of 0.455 MPa. The samples were heated from room temperature to 100 °C at a ramp rate of 2 °C/min. The HDT indicated the temperature at which a deflection of 0.25 mm occurred.

### 2.13. Melt Flow Index (MFI)

MFI of various blends was obtained by a Melt Flow Indexer (Srsy1, China) at 150 °C with a load of 2.16 Kg. The test was carried out according to ASTM D1238. Each sample was tested five times and the average value was given.

## 3. Results and Discussion

### 3.1. Mechanical Properties

Table 1 summarizes the mechanical properties of the binary and ternary blends. The tensile strength of TPS/PBS 60/40 was 15.4 MPa; the elongation at break was only 4.3%. The result shows that TPS/PBS 60/40 is a rigid polymer and deforms in a brittle fashion. On the contrary, TPS/PBAT 60/40 showed better flexibility with elongation over 80.6%, while the strength was poor and only 5.6 MPa. Interestingly, the ternary blends showed an advantage in tensile properties over the binary blends. The elongation at break and tensile strength of the TPS/PBS/PBAT 60/30/10 blend reached 25.6% and 12.4 MPa, it was six times and twice over that of TPS/PBS 60/40, respectively. For TPS/PBAT/PBS 60/20/20 blend, the elongation at break was increased to 38.4% and the tensile strength was 100% higher than TPS/PBAT 60/40. The tensile strength and the elongation at break of TPS/PBS/PBAT 60/10/30 were 10.3 MPa and 68.4%, which showed a balance property both in tensile strength and the elongation at break. Obviously, the tensile strength improvement is directly related to the amount of PBS in the blends, while the elongation at break increased with the addition of PBAT. Ren et al. reported a similar result in TPS/PLA/PBAT blends and found that with PLA as a reinforcer, and PBAT as a toughener, the TPS/PBAT/PLA50/40/10 component could achieve a tensile strength of 12 MPa with a guaranteed elongation at break of 15%.

The mechanical properties suggest that the interplay of the PBS and PBAT minor phases in the TPS matrix provided TPS/PBAT/PBS ternary blends a good balance of mechanical properties, which is a potential substitute for non-biodegradable polymers in the packaging.

### 3.2. Thermal and Crystallization Behavior of Binary and Ternary Blends

**Figure 2:**
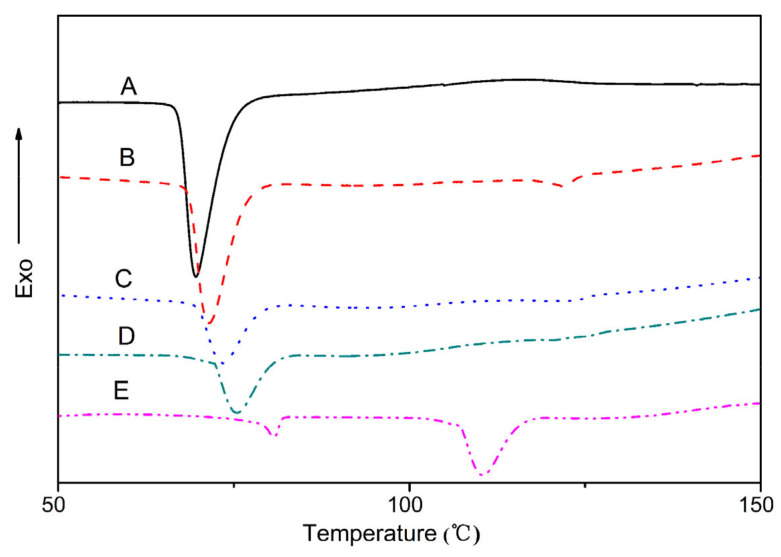
DSC thermograms at cooling rate 10 °C/min for all blends melted at 150 °C for 3 min: (A) TPS/PBS 60/40; (B) TPS/PBS/PBAT (60/30/10); (C) TPS/PBS/PBAT 60/20/20; (D) TPS/PBS/PBAT 60/10/30; (E) TPS/PBAT 60/40.

Figure 2 shows the DSC cooling thermograms at a cooling rate of 5 °C/min for the binary and ternary blends after being melted at 150 °C for 3 min, Temperature corresponding to the exothermic peak indicates the cold crystallization temperature (Tc) of the binary and ternary blends. All blends had obvious exothermic peaks corresponding to phase crystallization. With the addition of plasticizers of glycerol and water, the crystalline structure of starch was commonly disturbed and existed in an amorphous state. The transition peaks at 69 °C in the TPS/PBS60/40 blend appeared that related to the crystallization temperature (T_c_) of PBS. For TPS/PBAT60/40 blend, the crystallization peak temperature (T_c_) was about 110 °C, which belonged to the crystallization temperature of PBAT. Comparing the curves of ternary blends with binary blends, it was found that there was no obvious PBAT crystal exothermic peak in the DSC curves of ternary blends. The result indicated that the crystallization of PBAT was restricted by TPS and PBS due to the good miscibility of PBAT and PBS. In addition, the T_c_ of ternary blends was shifted to a higher temperature with the PBAT addition. It can be explained that TPS and PBS restrained PBAT spontaneous nucleation in the blend, and the PBAT acted as a nucleating agent for the TPS/PBS with the migration of heterogeneity from PBAT to PBS, which improved the crystallization temperature in the PBS-contained blend. The result was in accordance with the previous report on the crystallization behavior of other PBAT-contained blends [18]. For the TPS-based blends, because the dilution effect of PBS melt reduces the amount of PBAT chain segments toward the growing crystals, the crystallization growth rate of PBAT decreased. We speculate that during the cooling process PBAT forms small-dispersed crystals, which act as nucleation sites of PBS. Consequently, the improvement in PBS crystallization may be attributed to the positive effect of PBAT on the nucleation of PBS.

**Figure 3:**
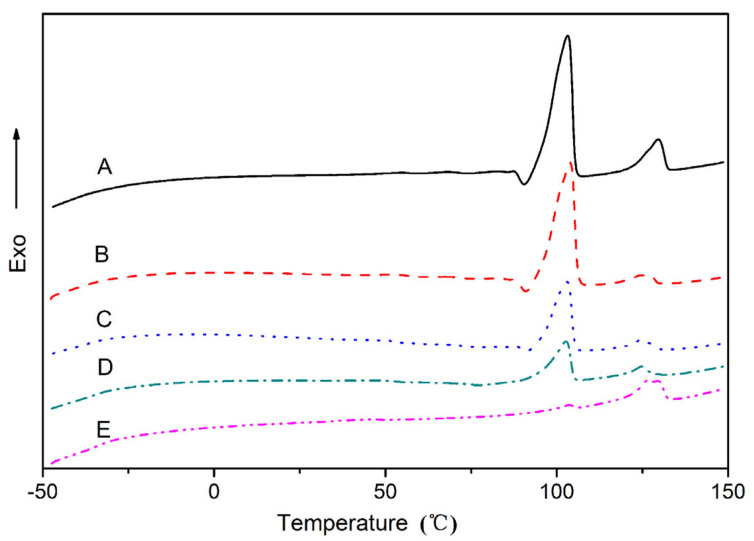
Second heating DSC thermograms for TPS/PBS and TPS/PBS/PBAT blends with different contents after cooling at 10 °C/min: (A) TPS/PBS 60/40; (B) TPS/PBS/PBAT (60/30/10); (C) TPS/PBS/PBAT 60/20/20; (D) TPS/PBS/PBAT 60/10/30; (E) TPS/PBAT 60/40.

Figure 3 resent the second heating thermograms of binary and ternary blends. The temperature corresponding to the heat absorption peak indicates the melting points temperature (T_m_). All DSC curves of PBS-contained blends show obvious double melting peaks upon crystallized non-isothermally at the given cooling rate. Many authors have explained double melting behavior by the melt recrystallization model. The exothermic dip between the double melting peaks was attributed to the recrystallization [19]. The crystalline melting point of PBS/PBAT blends became higher with the increased amount of PBAT. In general, the addition of PBAT is conducive to the heterogeneous nucleation of PBS, and the crystallization of PBS at a higher melting temperature is conducive to enhancing the crystal perfection and improving the melting point of the TPS/PBAT blend. Such an increase in the chemical potential of the crystallizable polymer was due to the partially miscible or compatible polymer, resulting in an increase in the melting point [20]. 

### 3.3. Dynamic Mechanical Analysis

**Figure 4:**
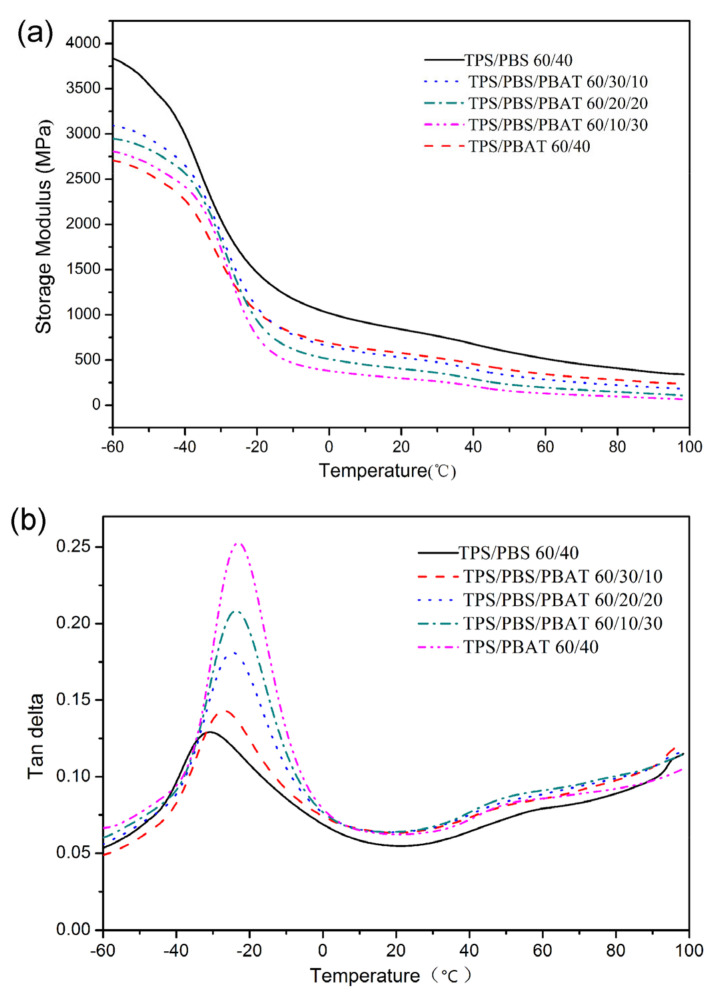
DMA thermogram of the binary and ternary blends: (**a**) Storage modulus versus tempera-ture; (**b**) Tan delta versus temperature.

Storage modulus (E′) is the energy stored and recovered in cyclic deformation. Figure 4a illustrates the temperature dependence of E′. For the binary blends, the storage modulus of TPS/PBS 60/40 is higher than that of TPS/PBAT 60/40 during the whole temperature range. The result indicated that PBS was higher in stiffness and had a greater ability to resist deformation than PBAT. For the ternary blends, a lower storage modulus was observed with higher content of PBAT. Furthermore, the material transformed into being elastic or conversely less permanently deformable with the addition of PBAT. The increased extent of onset points in TPS/PBS/PBAT 60/10/30 was higher as compared to the other blends, which was consistent with the results of DSC. Compared with binary blends, the onset increase point of ternary blends in storage modulus curves was higher. It indicated some interaction occurred among the different composites in the ternary blend and led to the cold crystallization at a higher temperature. All the binary and ternary blends had a single transition peak corresponding to the glass transition temperature, which indicated all TPS-based blends were partially compatible. In addition, The T_g_ of the ternary blends shifted to higher temperatures with a higher content of PBAT. This is consistent with the law that the glass transition temperature of compatible polymer blends is between the components and is related to the weight fraction of each component; tan δ is defined as the ratio of the loss modulus to the storage modulus, reflecting the viscoelastic tendency of the material. The addition of PBAT significantly increased the tan δ value of the blend around the glass transition temperature, indicating that the flexible molecular segment of PBAT promoted the viscous flow of the blend under external force. PBAT showed better miscibility with TPS/PBS blend, indicating that there is an interfacial interaction between them. The result further confirmed the early findings of the thermal effect of the transesterification reaction between PBS and PBAT [21].

### 3.4. X-ray Diffraction Analysis

**Figure 5:**
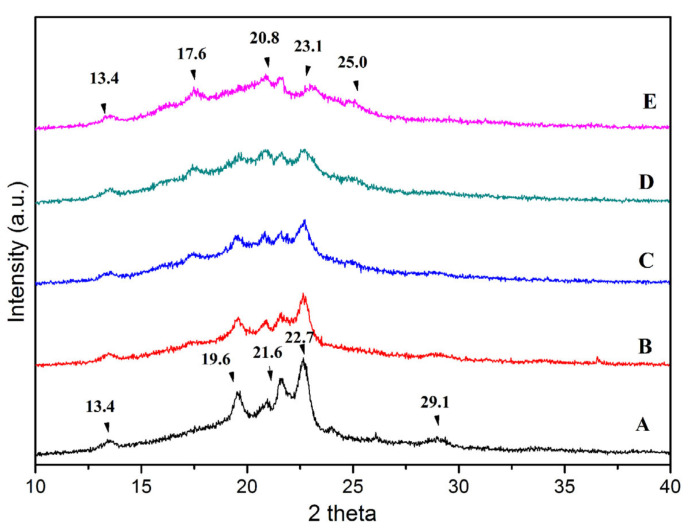
XRD pattern of binary and ternary blends: (A) TPS/PBS 60/40; (B) TPS/PBS/PBAT 60/30/10; (C) TPS/PBS/PBAT 60/20/20; (D) TPS/PBS/PBAT 60/10/30; (E) TPS/PBAT 60/40.

XRD is adopted to investigate the differences in crystal types or grain sizes among TPS-based blends. The intensity of the diffraction peak in XRD is always proportional to the peak area. Figure 5 shows the X-ray diffractograms of binary and ternary blends. The peaks at 2theta of 13.4° and 19.6° were characteristic peaks of TPS, which correspond to the V_H_ type amylose crystal [22]. The strong peaks at 2theta = 21.6° and 22.7° (Figure 5A) belong to the PBS crystallinity. It was consistent with the result reported by Kunyu Zhang [23]. The 2theta at 17.5°and 23.1° represented the crystalline structure of PBAT for TPS/PBAT blends. This was in accordance with the result of Raquezetal [24]. The diffraction peaks at 13.4°, 17.5°, 19.6°, 21.6°, 22.7°, and 23.1° were seen in all ternary blends, indicating all ternary blends had a similar crystalline structure. However, the diffraction peak became weaker and some new peaks appeared with the increased content of PBAT. This indicates that the addition of PBAT is conducive to PBS crystallization, but it affects the crystal structure of PBS. It was reported that the transesterification reaction that occurs widely in PBAT/PBS blends not only improved the interfacial compatibility of the blends but also changed their original crystallization types [21]. In addition, the PBS crystal peak in the ternary blend widened gradually due to the decreased crystal size caused by the heterogeneous nucleation crystallization of PBS.

### 3.5. Rheological Properties

**Figure 6:**
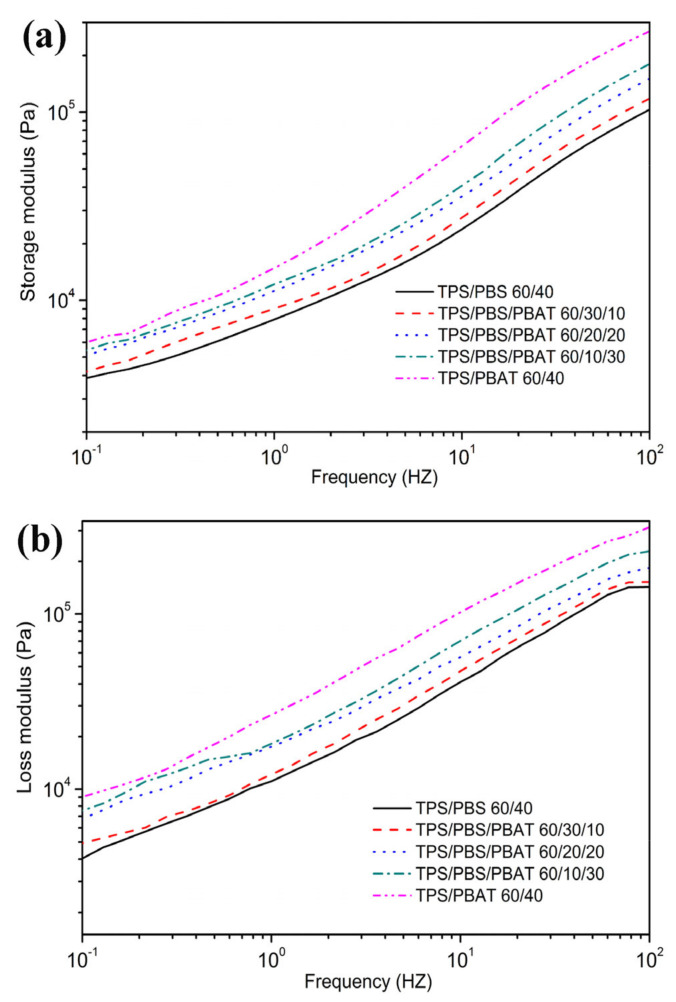
Storage modulus (**a**) and loss modulus (**b**) versus frequency curves of the binary and ternary blends at 140 °C.

Figure 6 detailed the storage modulus (G′) and loss modulus (G″) of the binary and ternary blends versus frequency. In this study, we found that the G′ and G″ of all samples enlarged monotonously with the increase in frequency. This result is in line with the basic laws of linear viscoelastic theory. In addition, G′ is always lower than G″, indicating all samples performed viscous behavior. For binary blends, TPS/PBAT60/40 exhibited higher G′ and G″ than that of the TPS/PBS60/40 blend during the whole angular frequency range. The result suggests that the molecular chain entanglement of PBAT was stronger than that of PBS in binary blends. For ternary blends, the G′ and G″ markedly increased with the increased amount of PBAT in the blends during the whole angular frequency range, which indicated the entanglement density increased with the increased amount of PBAT in the blends. Generally, the entanglement density of the blend depends on the existing compatibility in the blends. TPS/PBS/PBAT60/10/30 blend had the highest G′ and G″ in all ternary blends, which means that the blend has the best interface compatibility. 

**Figure 7:**
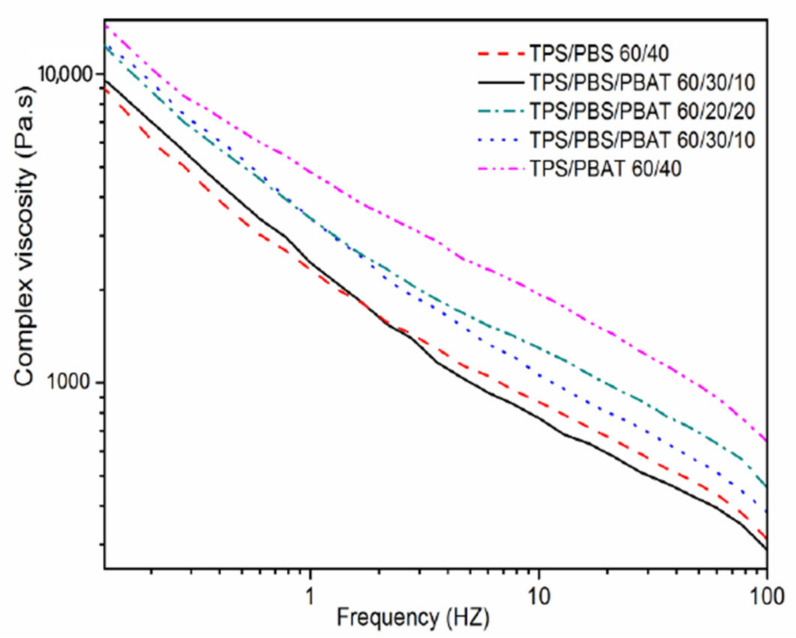
Complex viscosity (η*) frequency curves of the binary and ternary blends at 140 °C.

Figure 7 showed the complex viscosity (η*) of the binary and ternary blends as a function of frequency at 140 °C the η* of all samples reduced with the increased frequency, showing the characteristic of pseudoplastic and shear thinning. During the lower frequency range, the ternary blends with higher amounts of PBAT had larger complex viscosity. The change in complex viscosity is attributed to the PBAT molecular chain entanglement with PBS molecular chain mediated by the transesterification product acting as a compatibilizer [21], which formed pseudo structures to withstand shear forces. Ralltry [25] et al. also reported a similar behavior in PLA/PBAT blends at lower frequencies and considered that the melt viscosity was increased because of the interaction between the polymers.

**Figure 8:**
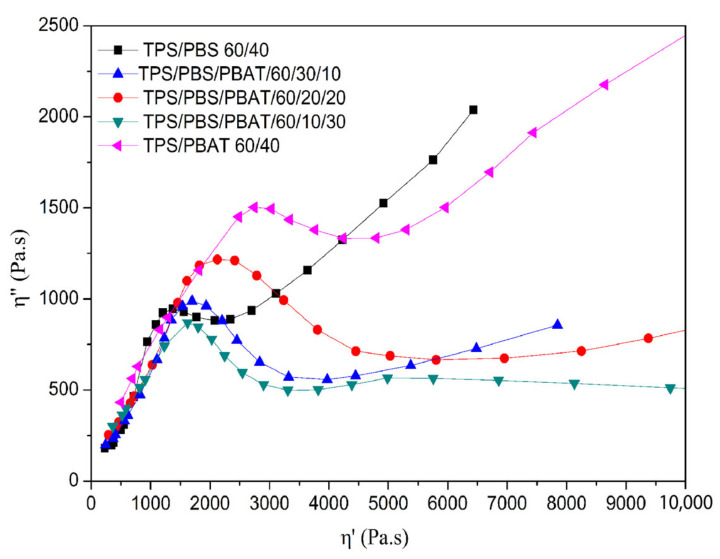
Cole–Cole plot of imaginary viscosity (η″) as a function of real viscosity (η′) for binary and ternary blends at 140 °C.

The Cole–Cole plot mainly describes the viscoelastic properties of the blends with a relaxation time distribution, and the plot was shown between imaginary viscosity (η″) and real viscosity (η′). In this study, the phase microstructure of the blends was also explored by the Cole–Cole plot. A single arc curve suggested the blend at the melt stage with phase homogeneity. When any deviation was observed from a single arc, the second relaxation and phase separation occurred in the blends to form the inhomogeneous morphology. Figure 8 depicts the Cole–Cole plots for the binary and ternary blends at 140 °C. For binary blends, a tail was observed on the right-hand side of the curve indicating that phase inversion occurred, indicating an inhomogeneous morphology formed. For the ternary blends, the tail gradually disappeared as the amount of PBAT increased. The second circular arc was observed on the right-hand side of the TPS/PBS/PBAT 60/10/30 blend curve, indicating the second relaxation mechanism happened. Obviously, the TPS/PBS/PBAT 60/10/30 was the most heterogeneous. The result confirmed that the further addition of PBAT helped to enhance the phases integrated into the blends. 

### 3.6. Phase Morphology of TPS Based Blends

**Figure 9:**
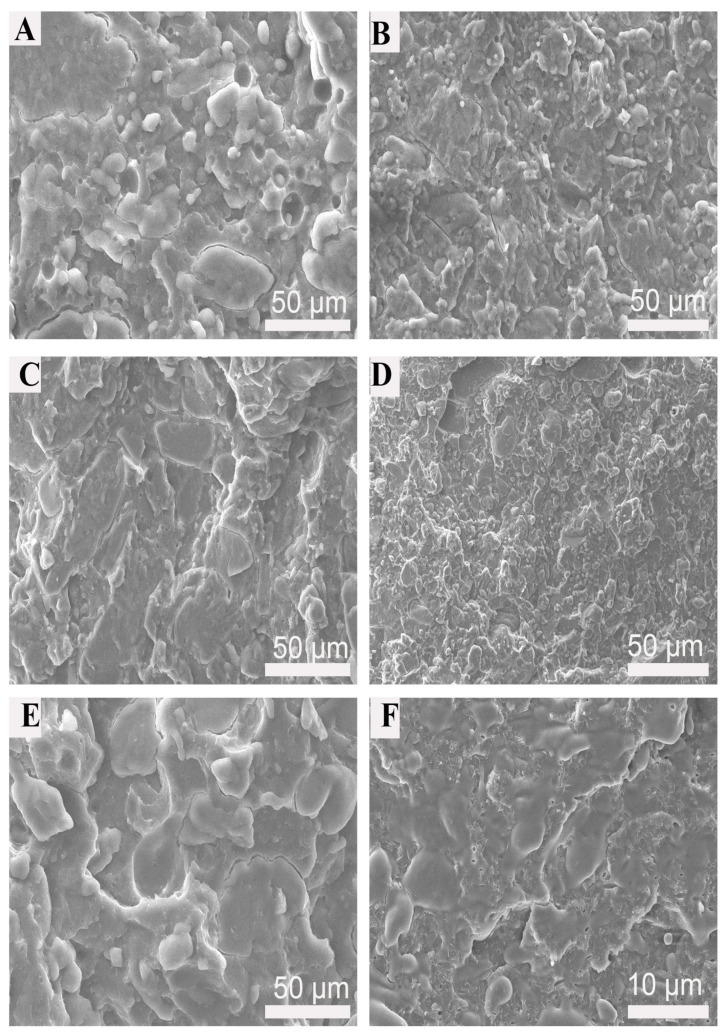
SEM images of cryofracture surface of the TPS based blends: (**A**) TPS/PBS 60/40; (**B**) TPS/PBS/PBAT 60/30/10; (**C**) TPS/PBS/PBAT 60/20/20; (**D**) TPS/PBS/PBAT 60/10/30; (**E**) TPS/PBAT 60/40; (**F**) TPS/PBS/PBAT 60/10/30 (high magnification of 5000).

Phase morphology plays a vital role in determining the blend properties, such as mechanical and thermal properties. The phase morphology of the blends depends on the second component, processing parameters, molecular weight of the virgin polymers and compatibility between the polymers. Figure 9 shows that TPS was partially plasticized in the binary blends and could not flow as a thermoplastic. For binary blends, the surface was smooth with an obvious phase interface, indicating poor compatibility and weak interfacial adhesion between the polyester and the starch. For ternary blends, starch granules melted and formed a continuous phase. PBS and PBAT domains are preferentially dispersed as spheres in the continuous TPS matrix with indistinct surfaces. With the increased content of PBAT, starch granules were distributed uniformly, and the ternary blends formed a typical mixture of core−shell morphology. Therefore, the TPS-based ternary blends had higher mechanical properties than the TPS-based binary blends. TPS/PBS/PBAT60/30/10 had the finest and most uniform morphology and enhanced the mechanical properties. 

### 3.7. Thermogravimetric Analysis (TGA)

**Figure 10:**
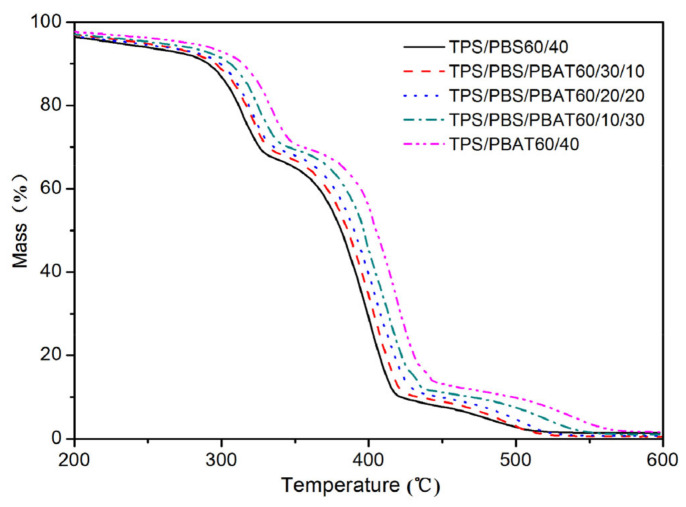
TGA curves of binary and ternary blends.

Figure 10 shows the thermal stability of TPS-based binary and ternary blends. All samples experienced the first slight loss of mass between 200 °C and 300 °C, due to the loss of water and glycerol molecules bound in the TPS. The slight increase in initial weight loss temperature compared to that reported by Pokhrel et al. [26] may be due to the different strengths of the hydrogen bonds between the plasticizers and starch molecules. The first major mass loss in the DTA curve occurs at around 320 °C, corresponding to the degradation of the starch molecular chains, which coincides with the thermal degradation temperature of TPS reported by Cai et al. [27]. We suggest that the addition of a bulking agent to enhance the interaction between the TPS phase and the polyester phase leads to a certain change in the thermal degradation temperature of TPS. As PBAT and PBS have similar thermal degradation temperatures, their thermal degradation occurs at around 400 °C, corresponding to the second major mass loss in the TGA curve. This is consistent with the data previously reported [28]. We define Tonset as the temperature corresponding to the sample mass loss when it reaches 5% and we found that the Tonset was above 300 °C for all samples, indicating that the TPS/PBS/PBAT blend has good thermal stability. In addition, the thermal stability of the blends increased with the increased content of PBAT, due to the improved compatibility between the polymer phases. The results of DSC and DMA further showed that the ternary blends were compatible. 

### 3.8. Heat Deflection Temperature

HDT is defined as the temperature where a material deflects 0.25 mm under 0.455 MPa load, representing the upper working temperature limit of a plastic. Table 2 reveals the HDT of all TPS-based blends. The HDT of the TPS/PBS 60/40 blend and TPS/PBAT 60/40 blend were 58.1 °C and 65.2 °C, respectively, in addition, an improvement of HDT from 59.4 to 63.5 °C was obtained when the content of PBAT increased from 10 wt% to 30 wt%. The HDT of ternary blends increases with the addition of PBAT, due to its higher thermal resistance. 

### 3.9. Melt Flow Index (MFI)

As a common processing parameter in actual production, the measurement of MFI has important practical significance. As illustrated in Table 3, the MFI of TPS/PBS60/40 and TPS/PBAT 60/40 were 2.15 g and 3.25 g per 10 min. The addition of PBAT improves the melt fluidity of TPS blends, the MFI of all TPS-based ternary blends was higher than those of binary polymers and TPS/PBS/PBAT 60/10/30 had the highest melt flow index of up to 7.35 g per 10 min. In general, the micro-morphology of blends, such as phase size and phase distribution, will affect their melt flow properties. TPS/PBS/PBAT 60/10/30 has the highest melt index because it forms the finest and uniform phase distribution (Figure 9). The result indicated that the TPS-based ternary blends had an advantage over TPS-based binary blends on flow behavior, which is beneficial for use as fillers or fiber-reinforced composites.

## 4. Conclusions

We successfully developed a fully biodegradable TPS/PBS/PBAT ternary blend with a high content of TPS. The TPS-based ternary blends exhibited unique synergistic mechanical properties. The uniform dispersion of PBS and PBAT minor phases greatly improves tensile strength and elongation at break. The TPS/PBS/PBAT60/10/30 blend formed a typical mixture of core−shell morphology and possessed the optimal balance in the strength of 10.3 Mpa and elongation over 68.4%. With the increased content of PBAT, phase morphology changes occurred in ternary blends, leading to decreased values of complex viscosity, storage modulus and loss modulus. In addition, the TPS/PBS/PBAT 60/10/30 blend shows excellent thermal resistance. The obtained results indicated that the ternary blends of TPS/PBS/PBAT showed potential application prospects in the packaging materials field. 

## Figures and Tables

**Table 1:** Mechanical properties of the binary and ternary blends.

Samples	Tensile Strength (MPa)	Elongation at Break (%)
TPS/PBS 60/40	15.4 ± 0.3 ^a^	4.3 ± 5.3 ^a,b^
TPS/PBS/PBAT60/30/10	12.4 ± 0.4 ^a^	25.6 ± 7.8 ^a,b^
TPS/PBS/PBAT60/20/20	11.3 ± 0.3 ^a^	38.4 ± 10.3 ^a,b^
TPS/PBS/PBAT60/10/30	10.3 ± 0.1 ^a^	68.4 ± 5.5 ^a,b^
TPS/PBAT60/40	5.6 ± 0.2 ^a^	80.6 ± 8.3 ^a,b^

^a,b^ Means with different letters under same column indicate a difference at 0.05 level by Tukey’s test.

**Table 2:** Heat deflection temperature (HDT).

Samples	HDT/°C
TPS/PBS 60/40	58.1 ± 0.2
TPS/PBS/PBAT60/30/10	59.4 ± 0.3
TPS/PBS/PBAT60/20/20	61.3 ± 0.5
TPS/PBS/PBAT60/10/30	63.5 ± 0.1
TPS/PBAT60/40	65.2 ± 0.1

**Table 3:** Melt Flow Index (MFI) of binary and ternary blends.

Samples	MFI (190 °C) g/10 min
TPS/PBS 60/40	2.15 ± 0.32
TPS/PBS/PBAT60/30/10	3.94 ± 0.46
TPS/PBS/PBAT60/20/20	4.30 ± 0.13
TPS/PBS/PBAT60/10/30	7.35 ± 0.21
TPS/PBAT60/40	3.25 ± 0.18

## Data Availability

Not applicable.
